# Mortality predictors in acute myocardial infarction: results from a single-center study in Saudi Arabia

**DOI:** 10.25122/jml-2024-0366

**Published:** 2024-11

**Authors:** Yasir Abdulmohsen Alzalabani, Bader Osama Sager, Hamzah Khalid Ibrahim, Faisal Mohammed Alnami, Yazeed Mosa Alharbi, Ammar Khalid Almatrafi, Ayat Roushdy

**Affiliations:** 1 College of Medicine, Taibah University, Medina, Kingdom of Saudi Arabia; 2 Epidemiology and Preventive Medicine Department, National Liver Institute (NLI), Menoufiya University, Shibin Al Kawm, Egypt; 3 Family and Community Medicine Department, College of Medicine, Taibah University, Medina, Kingdom of Saudi Arabia

**Keywords:** myocardial infarction, Saudi Arabia, Middle East, predictors of mortality, MI, myocardial infarction, STEMI, ST-segment elevation myocardial infarction, NSTEMI, non-ST-elevation myocardial infarction, CAD, coronary artery disease, SPACE, Saudi Project for Assessment of Coronary Events, ECG, electrocardiography, LDL, low-density lipoprotein, KSA, Kingdom of Saudi Arabia, SD, standard deviation, CK-MB, creatine kinase-myocardial band, BUN, blood urea nitrogen, DM, diabetes mellitus, HTN, hypertension, PCI, percutaneous coronary intervention, KAMIR-NIH, Korea Acute Myocardial Infarction Registry–National Institute of Health, TG, triglycerides, HDL, high-density lipoprotein, HRs, hazard ratios, IHD, ischemic heart disease, BMI, body mass index

## Abstract

Acute myocardial infarction (AMI) is a leading cause of morbidity and mortality worldwide. Risk factors of mortality in patients with AMI have been widely investigated, identifying older age and heart failure as common contributors. This study aimed to determine risk factors and explore predictors associated with higher mortality among patients with AMI. A retrospective study was conducted at a cardiac center in western Saudi Arabia (KSA) between January 1, 2023, and September 1, 2023. Inclusion criteria comprised patients with a confirmed diagnosis of AMI. Exclusion criteria included patients younger than 18 and those with incomplete diagnostic or follow-up data. A data collection form was generated, including all possible factors associated with mortality among patients with AMI. The study included 851 MI patients with a mean age of 58.78 years, primarily male participants. Survival analysis based on the days of hospitalization revealed that 30-day and 60-day survival rates post-hospitalization were 66.8% and 33.4%, respectively. Patients with acute MI of the anterior wall or other specific sites demonstrated significantly higher risks of mortality compared to those with unspecified acute MI. Elevated creatine kinase-myocardial band (CK-MB) levels and blood urea nitrogen (BUN) were also significantly associated with increased mortality risk. The findings highlighted an association between mortality and diabetes mellitus (DM) and transmural MI of the anterior wall. Significant differences between surviving and deceased patients were observed in several factors, including troponin, CK-MB, low-density lipoprotein (LDL), BUN, creatinine levels, age, and hospital stay duration.

## INTRODUCTION

Acute myocardial infarction (AMI), which includes ST-segment elevation myocardial infarction (STEMI) and non-ST-elevation myocardial infarction (NSTEMI), is a leading cause of morbidity and mortality worldwide. Acute myocardial infarction remains a significant cause of adult mortality despite earlier detection and advancements in management [1]. Numerous studies from Western and Middle Eastern regions have identified older age, heart failure, and previous history of stroke and coronary artery bypass graft as key predictors of mortality among patients with AMI [1-5].

Coronary artery disease (CAD), the primary underlying condition for AMI, is influenced by both modifiable risk factors—such as hypercholesterolemia, hypertension, diabetes mellitus (DM), smoking, obesity, psychological stress, and physical inactivity—and non-modifiable factors, including advanced age, male sex, family history, and racial predispositions [6]. Middle Eastern populations, in particular, frequently present with multiple risk factors, with hypertension, smoking, and diabetes mellitus (DM) being the most prevalent [7]. CAD in the Middle East tends to manifest approximately 10 years earlier than in global populations. Although there is a lack of information on the precise epidemiology of CAD in Saudi Arabia, a prevalence of 5.5% among people between the ages of 30 and 70 was reported in 2004 [8]. The Saudi Project for Assessment of Coronary Events (SPACE) registry further highlighted the characteristics and prevalence of CAD risk factors in Saudi Arabia, reporting ischemic heart disease (IHD) in 32% of patients and identifying DM (56%), hypertension (48%), smoking (39%), and hyperlipidemia (31%) as the most common risk factors [9]. Saudi Arabia has undergone significant socioeconomic growth over recent years, and its social and nutritional statuses now match those of Western nations. As a result, there has been a rise in the incidence of CAD among the Saudi population [10].

A lack of high-quality studies on the predictors of mortality among patients with AMI in the Saudi population motivated us to conduct the current study. We aimed to determine the risk factors and explore the predictors associated with higher mortality among patients with AMI admitted to a single cardiac center in western Saudi Arabia between January 1^st^, 2023, and September 1^st^, 2023.

## MATERIAL AND METHODS

This retrospective cross-sectional observational study was conducted to analyze the experience of a single cardiac center in the western region of Saudi Arabia. Temporary electronic access to patient files was granted to facilitate data collection. The study included all patients with a confirmed diagnosis of acute myocardial infarction admitted to the cardiac center between January 1, 2023, and September 1, 2023. A total of 862 participants were included in the study.

Inclusion criteria required participants to have a confirmed diagnosis of AMI based on symptoms of ischemia, serial electrocardiograms (ECGs), and serial cardiac marker measurements. Additionally, immediate coronary angiography was required for patients with ST-segment elevation myocardial infarction (STEMI) or complications (e.g., persistent chest pain, hypotension, markedly elevated cardiac markers, or unstable arrhythmias). For patients with non-ST-elevation myocardial infarction (NSTEMI) without complications, delayed coronary angiography within 24 to 48 hours was considered sufficient. Exclusion criteria included patients younger than 18 or with incomplete diagnostic or follow-up data.

A data collection form was generated that included potential risk factors for AMI and factors that could be associated with mortality among AMI patients. The data collection form included sociodemographic data (age, gender, residence, nationality, smoking status, and family history of cardiac diseases), clinical data (weight, height, body mass index [BMI], history of diabetes mellitus, history of hypertension, type of ischemic heart disease, previous history of myocardial infarction (MI) and ischemic heart disease, discharge status, and hospital stay), laboratory results (troponin, creatine kinase- myocardial band (CK-MB), triglycerides, low-density lipoprotein [LDL], high-density lipoprotein [HDL], blood urea nitrogen [BUN], and creatinine). Data were collected from the electronic records of all included AMI patients.

### Statistical analysis

Data analysis was performed using RStudio (R version 4.3.1). Categorical variables were presented as frequencies and percentages, and numerical variables as means with standard deviations (SD). The differences between deceased and surviving patients in terms of categorical variables were assessed using Pearson's chi-square test or Fisher's exact test as applicable. For continuous variables, differences were assessed using a Wilcoxon rank sum test. Survival probabilities were visualized using Kaplan–Meier plots. Risk factors for death were initially explored by constructing univariable Cox proportional hazard models using the primary outcome variable (death) as the dependent variable and the length of hospital stay as a time-to-event variable. The significantly associated variables from the univariable analysis were used as independent variables in a multivariable hazard regression model. Results were expressed as hazard ratios (HRs) and their respective 95% confidence intervals (95% CIs). Statistical significance was considered at *P* < 0.05.

## RESULTS

### Sociodemographic characteristics

Data were initially collected from 862 patients. However, two patients aged <18 years and nine patients with a missing discharge status (death was a primary outcome) were excluded. Therefore, the final sample included 851 patients with AMI from a single cardiac center in the western region of Saudi Arabia. The majority were men (80.3%). The mean length of hospital stay was 4.39 ± 8.06 days, and the mean age was 59.08 ± 12.54 years. More than half of the patients were of Saudi nationality (56.5%), and the majority resided within the Medina province (96.0%). Among the patients, 54.9% were non-smokers, and 89.6% had no family history of cardiac diseases. The mean weight and height were 76.16 ± 14.40 kg and 167.43 ± 8.43 cm, respectively, while the mean BMI was 27.13 ± 4.79 kg/m^2^ (Table 1).

**Table 1:** Demographic characteristics of participants

Characteristic	*P* value
Missing	Surviving group *n* = 804 *n* (%)	Deceased group *n* = 47 *n* (%)	*n* = 851 *n* (%)
**Gender**	0 (0%)				0.786
Male		646 (80.3%)	37 (78.7%)	683 (80.3%)	
Female		158 (19.7%)	10 (21.3%)	168 (19.7%)	
**Discharge status**	0 (0%)				<0.001
Home/Other		783 (97.4%)	0 (0.0%)	783 (92.0%)	
Discharge transfer to other healthcare accommodation		3 (0.4%)	0 (0.0%)	3 (0.4%)	
Left against medical advice		18 (2.2%)	0 (0.0%)	18 (2.1%)	
Mortality		0 (0.0%)	47 (100.0%)	47 (5.5%)	
**Nationality**	0 (0%)				0.280
Saudi		458 (57.0%)	23 (48.9%)	481 (56.5%)	
Non-Saudi		346 (43.0%)	24 (51.1%)	370 (43.5%)	
**Residence**	5 (0.6%)				0.114
Inside Medina province		769 (96.2%)	43 (91.5%)	812 (96.0%)	
Outside Medina province		30 (3.8%)	4 (8.5%)	34 (4.0%)	
**Smoking status**	58 (6.8%)				0.012
Current smoker		266 (35.2%)	6 (15.8%)	272 (34.3%)	
Ex-smoker for more than 2 years		78 (10.3%)	8 (21.1%)	86 (10.8%)	
Non-smoker		411 (54.4%)	24 (63.2%)	435 (54.9%)	
**Family history of cardiac diseases**	91 (11%)				0.163
No		646 (89.2%)	35 (97.2%)	681 (89.6%)	
Yes		78 (10.8%)	1 (2.8%)	79 (10.4%)	
**Quantitative variables**		**Mean ± SD**	**Mean ± SD**	**Mean ± SD**	***P* value**
Age		58.78 ± 12.57	64.28 ± 11.03	59.08 ± 12.54	<0.001
Weight		76.32 ± 14.46	72.89 ± 12.83	76.16 ± 14.40	0.160
Height		167.48 ± 8.51	166.37 ± 6.41	167.43 ± 8.43	0.287
BMI		27.16 ± 4.80	26.39 ± 4.40	27.13 ± 4.79	0.396
Length of hospital stay (days)		4.19 ± 7.82	7.77 ± 11.01	4.39 ± 8.06	0.027

Most patients were discharged home (92.0%), 2.1% left against medical advice, and 0.4% were transferred to other healthcare institutions. There was a 5.5% mortality recorded. There were no significant differences in gender distribution between deceased and surviving patients (*P* = 0.786), with the majority being men in both groups. Length of hospital stay and age showed statistically significant differences between survivors and deceased patients (*P* = 0.027 and *P* < 0.001, respectively), with deceased patients having a longer hospital stay (7.77 ± 11.01 days) and being older (64.28 ± 11.03 years) compared to survivors (4.19 ± 7.82 days and 58.78 ± 12.57 years, respectively). Additionally, smoking status was significantly associated with mortality (*P* = 0.012), with a higher proportion of deceased patients identified as ex-smokers who had quit smoking for more than two years (21.1%). Other variables, including nationality, residence, family history of cardiac diseases, weight, height, and BMI, were not significantly different between surviving and deceased patients (all *P* values > 0.05, Table 1).

### Clinical history and laboratory parameters

Hypertension was prevalent among the patients, with the majority having this condition, but it showed no significant difference between deceased and surviving groups (*P* = 0.372). However, diabetes was significantly associated with mortality (*P* = 0.013), with a higher percentage of deceased patients having diabetes (73.2%) compared to survivors (26.8%). Although a history of IHD was not significantly associated with mortality (*P* = 0.191), the type of AMI was significantly associated with mortality (*P* = 0.002), with acute transmural MI of the anterior wall observed in a higher proportion of deceased patients (47.8%). Among the laboratory parameters, troponin (*P* = 0.008), CK-MB (P < 0.001), LDL (*P* = 0.029), BUN (*P* < 0.001), and creatine (*P* < 0.001) levels were significantly higher in deceased patients. No significant differences were observed for triglycerides (TG) and HDL levels between the two groups (*P* > 0.05, Table 2).

**Table 2:** Clinical history and laboratory parameters of participants

Characteristic	Missing	Death		Overall	*P* value
		No *n* = 804 *n* (%)	Yes *n* = 47 *n* (%)	*n* = 851 *n* (%)	
**Hypertension**	50 (5.9%)				0.372
No		388 (51.1%)	18 (43.9%)	406 (50.7%)	
Yes		372 (48.9%)	23 (56.1%)	395 (49.3%)	
**Diabetes**	43 (5.1%)				0.013
No		358 (46.7%)	11 (26.8%)	369 (45.7%)	
Yes		409 (53.3%)	30 (73.2%)	439 (54.3%)	
**History of IHD**	60 (7.1%)				0.191
No		584 (77.6%)	26 (68.4%)	610 (77.1%)	
Yes		169 (22.4%)	12 (31.6%)	181 (22.9%)	
**Type of AMI**	5 (0.6%)				0.002
Acute MI, unspecified		159 (19.9%)	1 (2.2%)	160 (18.9%)	
Acute subendocardial MI		211 (26.4%)	13 (28.3%)	224 (26.5%)	
Acute transmural MI of anterior wall		215 (26.9%)	22 (47.8%)	237 (28.0%)	
Acute transmural MI of the inferior wall		174 (21.8%)	8 (17.4%)	182 (21.5%)	
Acute transmural MI of another site		41 (5.1%)	2 (4.3%)	43 (5.1%)	
**Quantitative variables**		**Mean ± SD**	**Mean ± SD**	**Mean ± SD**	***P* value**
**Troponin**		20.66 ± 203.96	23.14 ± 55.97	20.78 ± 199.49	0.008
**CK-MB**		75.76 ± 134.98	238.35 ± 241.82	83.33 ± 145.59	<0.001
**TG**		1.67 ± 2.92	1.43 ± 0.88	1.66 ± 2.86	0.499
**LDL**		3.51 ± 22.37	22.77 ± 114.31	4.32 ± 31.97	0.029
**HDL**		1.33 ± 7.21	0.94 ± 0.37	1.32 ± 7.06	0.185
**BUN**		6.77 ± 6.91	18.95 ± 12.41	7.35 ± 7.71	<0.001
**Creatine**		102.07 ± 79.78	290.92 ± 189.31	111.13 ± 96.79	<0.001

IHD, Ischemic heart disease; MI, Myocardial infarction; CK-MB, Creatine kinase-myocardial band; LDL, Low-density lipoprotein; BUN, Blood urea nitrogen; TG, Triglycerides; HDL, High-density lipoprotein; BMI, Body mass index

### Survival analysis and predictors of mortality

**Figure 1:**
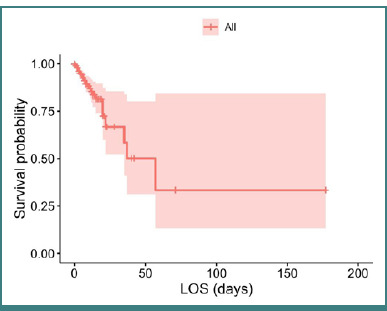
Kaplan–Meier curve depicting the survival probability of patients with MI

Survival analysis based on the days of hospitalization revealed that the 30-day survival rate post-hospitalization was 66.8% (95% CI, 52.2–85.5), and the 60-day survival rate was 33.4% (95% CI, 13.2–84.5; Figure 1).

In the multivariable Cox proportional hazard analysis, several key risk factors for mortality among patients with AMI were statistically significant. The specific type of AMI exhibited significant associations, with patients experiencing acute transmural MI of the anterior wall and acute transmural MI of another site demonstrating higher risks of mortality compared to those with unspecified acute MI (HR = 11.4, 95% CI, 1.30–101; *P* = 0.028, and HR = 16.3, 95% CI, 1.20–22; *P* = 0.036, respectively). Elevated levels of CK-MB were also significantly associated with an increased risk of death (HR = 1.00, 95% CI, 1.00– 1.00; *P* < 0.001). Furthermore, elevated BUN levels were significantly associated with a higher risk of mortality (HR = 1.05, 95% CI, 1.00–1.11; *P* = 0.032; Table 3).

**Table 3:** Cox proportional hazard regression analysis of mortality risk factors in patients with myocardial infarction

Characteristic	Univariable			Multivariable		
	HR	95% CI	*P* value	HR	95% CI	*P* value
**Gender**						
Male	Reference	Reference				
Female	1.08	0.54–2.18	0.826			
**Age**	1.02	1.00–1.05	0.053			
**Nationality**						
Saudi	Reference	Reference				
Non-Saudi	1.24	0.69–2.21	0.468			
**Residence**						
Inside Medina province	Reference	Reference				
Outside Medina province	1.95	0.69–5.47	0.205			
**Smoking status**						
Current smoker	Reference	Reference				
Ex-smoker for more than 2 years	2.80	0.95–8.22	0.061			
Non-smoker	1.91	0.77–4.71	0.160			
**Family history of cardiac diseases**						
No	Reference	Reference				
Yes	0.30	0.04–2.19	0.235			
**BMI**	0.99	0.91–1.07	0.726			
**Hypertension**						
No	Reference	Reference				
Yes	1.44	0.77–2.70	0.255			
**Diabetes**						
No	Reference	Reference				
Yes	1.72	0.86–3.47	0.128			
**History of IHD**						
No	Reference	Reference				
Yes	1.49	0.75–2.97	0.261			
**Type of AMI**						
Acute MI, unspecified	Reference	Reference		Reference	Reference	
Acute subendocardial MI	7.92	1.03–61.0	0.047	5.11	0.48–54.9	0.178
Acute transmural MI of anterior Wall	12.9	1.73–95.5	0.013	11.4	1.30–101	0.028
Acute transmural MI of the inferior wall	7.99	0.99–64.6	0.051	7.17	0.76–67.3	0.085
Acute transmural MI of another site	8.11	0.73–90.5	0.089	16.3	1.20–223	0.036
**Troponin**	1.00	1.00–1.00	0.997			
**CK-MB**	1.00	1.00–1.00	<0.001	1.00	1.00–1.00	<0.001
**TG**	0.91	0.60–1.37	0.642			
**LDL**	1.01	1.00–1.01	<0.001	1.00	1.00–1.01	0.095
**HDL**	0.88	0.29–2.68	0.817			
**BUN**	1.03	1.02–1.04	<0.001	1.05	1.00–1.11	0.032
**Creatine**	1.00	1.00–1.01	<0.001	1.00	1.00–1.00	0.099

## DISCUSSION

This cross-sectional study aimed to identify the predictors associated with higher mortality among AMI patients admitted to a cardiac center in the western region of Saudi Arabia from January 1^st^, 2023, to September 1^st^, 2023. We observed that the length of hospital stays and the age of patients were significantly different between survivors and deceased patients, with deceased patients having longer hospital stays and being older than survivors. These data agree with a previous US study examining patients undergoing primary percutaneous coronary intervention (PCI) for STEMI between 2002 and 2011 that noted the association between the length of hospital stay and mortality [11].

Other variables, including nationality, residence, family history of cardiac diseases, weight, height, BMI, and hypertension, did not have statistically significant differences between surviving and deceased patients, which is in line with the results of an earlier study that included 536 patients with AMI who underwent primary PCI in Japan between April 2010 and July 2016 [12]. However, there was no significant difference in HTN levels between deceased and surviving patients, likely due to the considerable proportion of patients overall having this condition (50.7% overall). Diabetes had a significant association with mortality, and a higher percentage of deceased patients had diabetes (73.2%) compared to survivors (26.8%).

Patients with acute transmural MI of the anterior wall comprised a higher proportion of deceased patients (47.8%). This aligns with the findings of a Korean study that included 13,104 patients with AMI between November 2011 and October 2015 [13]. Troponin, CK-MB, LDL, BUN, and creatine levels were significantly higher in the deceased group compared with the surviving group. The Korean study also highlighted these laboratory markers as risk factors in patients with AMI [13]. No significant differences between the two groups were observed in TG and HDL levels, consistent with previous reports [12]. However, the history of IHD, smoking, and renal impairment parameters did not demonstrate a statistically significant association with mortality in patients with AMI.

### Study limitations

The lack of clinical information is a limitation of this study. Factors such as the discharge status exhibited a highly significant difference, with no instances of mortality among patients discharged to other healthcare accommodations or against medical advice, contrasting sharply with the 100% mortality rate for those who died during the hospital stay. The absence of detailed patient histories, particularly for deceased individuals, limits the ability to identify additional mortality predictors. To address this, we recommend that future studies incorporate information obtained from the families of deceased patients to explore potential contributing factors. Most of this study’s limitations were due to the absence of post-mortality data. Furthermore, future studies should consider collecting additional clinical data, including Killip class, pre- and post-percutaneous coronary intervention, and laboratory findings, including glucose level, C-reactive protein, and glomerular filtration rate.

## CONCLUSION

This study assessed the predictors of mortality in patients with MI admitted to a single center and showed that DM and transmural MI of the anterior wall were associated with mortality among patients. HTN showed no significant difference between deceased and surviving patients. Regarding the laboratory markers, troponin, CK-MB, LDL, BUN, and creatinine showed significant differences between surviving and deceased patients, while no significant differences were observed for TG and HDL. Both age and length of hospital stay showed significant differences between surviving and deceased patients, with deceased patients having a longer hospital stay and being older. This study recommends early preventive and screening programs, as well as patient and community education, due to the increased mortality among the elderly population. Also, it supports the importance of controlling DM as a major risk factor for mortality. Future studies should consider the increase of troponin, CK-MB, LDL, BUN, and creatinine as risk factors for MI mortality.

## Data Availability

This article was published with the support granted by the project entitled "Net4SCIENCE: Applied doctoral and postdoctoral research network in the fields of smart specialization Health and The data used in this study are available to the corresponding author upon reasonable request.
